# Synaptic mitochondrial dysfunction and septin accumulation are linked to complement-mediated synapse loss in an Alzheimer’s disease animal model

**DOI:** 10.1007/s00018-020-03468-0

**Published:** 2020-02-07

**Authors:** Balázs A. Györffy, Vilmos Tóth, György Török, Péter Gulyássy, Réka Á. Kovács, Henrietta Vadászi, András Micsonai, Melinda E. Tóth, Miklós Sántha, László Homolya, László Drahos, Gábor Juhász, Katalin A. Kékesi, József Kardos

**Affiliations:** 1 ELTE NAP Neuroimmunology Research Group, Department of Biochemistry, Institute of Biology, ELTE Eötvös Loránd University, Budapest, Hungary; 2 Laboratory of Proteomics, Institute of Biology, ELTE Eötvös Loránd University, Budapest, Hungary; 3 Department of Biochemistry, Institute of Biology, ELTE Eötvös Loránd University, Budapest, Hungary; 4 Molecular Cell Biology Research Group, Institute of Enzymology, Research Centre for Natural Sciences, Hungarian Academy of Sciences Centre of Excellence, Budapest, Hungary; 5 Department of Biophysics and Radiation Biology, Semmelweis University, Budapest, Hungary; 6 MS Proteomics Research Group, Institute of Organic Chemistry, Research Centre for Natural Sciences, Budapest, Hungary; 7 Institute of Biochemistry, Biological Research Centre, Szeged, Hungary; 8 CRU Hungary Ltd., Göd, Hungary; 9 Department of Physiology and Neurobiology, Institute of Biology, ELTE Eötvös Loránd University, Budapest, Hungary

**Keywords:** Synaptic pruning, Alzheimer’s disease, Complement C1q, Fluorescence-activated synaptosome sorting, Mitochondrial dysfunction, Septins

## Abstract

Synaptic functional disturbances with concomitant synapse loss represent central pathological hallmarks of Alzheimer’s disease. Excessive accumulation of cytotoxic amyloid oligomers is widely recognized as a key event that underlies neurodegeneration. Certain complement components are crucial instruments of widespread synapse loss because they can tag synapses with functional impairments leading to their engulfment by microglia. However, an exact understanding of the affected synaptic functions that predispose to complement-mediated synapse elimination is lacking. Therefore, we conducted systematic proteomic examinations on synaptosomes prepared from an amyloidogenic mouse model of Alzheimer’s disease (APP/PS1). Synaptic fractions were separated according to the presence of the C1q-tag using fluorescence-activated synaptosome sorting and subjected to proteomic comparisons. The results raised the decline of mitochondrial functions in the C1q-tagged synapses of APP/PS1 mice based on enrichment analyses, which was verified using flow cytometry. Additionally, proteomics results revealed extensive alterations in the level of septin protein family members, which are known to dynamically form highly organized pre- and postsynaptic supramolecular structures, thereby affecting synaptic transmission. High-resolution microscopy investigations demonstrated that synapses with considerable amounts of septin-3 and septin-5 show increased accumulation of C1q in APP/PS1 mice compared to the wild-type ones. Moreover, a strong positive correlation was apparent between synaptic septin-3 levels and C1q deposition as revealed via flow cytometry and confocal microscopy examinations. In sum, our results imply that deterioration of synaptic mitochondrial functions and alterations in the organization of synaptic septins are associated with complement-dependent synapse loss in Alzheimer’s disease.

**Electronic supplementary material:**

The online version of this article (10.1007/s00018-020-03468-0) contains supplementary material, which is available to authorized users.

## Introduction

Alzheimer’s disease (AD) is characterized by excessive and persistent synapse loss, which increases during disease progression and later accompanied by widespread neuronal cell death that altogether lead to gradual deterioration of memory and other cognitive functions [1]. Importantly, the extent of synapse loss shows a considerably strong positive correlation with impairments in cognitive functions [2]. The underlying pathology in overabundant synapse loss is not fully deciphered yet; however, accumulation of toxic amyloid oligomers (formed from amyloid-beta (Aβ) 1–40 and 1–42 peptides) is widely recognized as a critical causative factor [3]. Microglial cells, the most abundant resident macrophages in the brain, and the complement cascade have gained increasing attention recently, focusing on the pathogenesis of AD. Activated “Janus-faced” microglia not just alleviate disease progression via clearance of Aβ [4] but also substantially contribute to neurodegeneration as they can amplify deleterious proinflammatory mechanisms [5] and engulf synapses [6]. Among microglial factors modulating neuronal functions, the complement cascade has a central role. Complement components are parts of the innate immune system involved in the rapid elimination of harmful and unnecessary cellular elements, e.g., invading pathogens and cellular debris [7], and also expressed in the central nervous system [8]. Notably, C1q, the initiator of the classical complement pathway, is dominantly synthesized by microglia in the brain [9]. Certain members of the complement cascade are known for more than a decade to be involved in the synaptic refinement in the developing visual system as they tag synapses for selective pruning [10]. Moreover, it has been demonstrated that the microglia–complement axis is substantially involved in pathological, excessive synaptic pruning in animal models of AD even in the early stages preceding the chronic neuroinflammatory condition [11]. Additional studies unveiled that beyond AD, complement conveys aberrant synapse loss in other disorders with exaggerated synaptic pruning, such as tauopathy-associated neurodegeneration [12], frontotemporal dementia [13], glaucoma [14], schizophrenia [15, 16], West Nile virus infection [17], hypoxic–ischemic brain damage [18], and traumatic brain injury [19].

We currently reported that complement-mediated synapse elimination is strongly dependent on the activation of local synaptic apoptotic-like processes in the healthy adult brain [20]. Nonetheless, the cellular events in the synaptic compartment that predispose to synaptic C1q-tagging and complement-mediated synapse loss under pathological conditions characterized by elevated synapse elimination remain elusive. To this end, in this study, we employed fluorescence-activated synaptosome sorting coupled to proteomics on the APPswe/PSEN1dE9 (APP/PS1) transgenic mouse model of AD. Double-transgenic APP/PS1 mice bearing mutant amyloid precursor protein (APP) and presenilin-1 (PS1) genes show gradual development of several well-known features of the disorder, e.g*.*, amyloid plaque deposition [21, 22], impaired synaptic plasticity [23], synapse loss [11], memory deficits, and behavioral abnormalities [24]. Previously, we described in the APP/PS1 mice the proteomic correlates of synaptic mitochondrial dysfunction [25], a widely accepted early cornerstone of AD development [26]. Noteworthy, critical accumulation of Aβ deposits appears relatively earlier in this mouse model than in the most common sporadic form of AD in affected elderly patients [22], enabling the more specific investigation of the detrimental effects of Aβ with less interference with the aging process. Most importantly, the crucial role of complement-dependent synapse loss in disease development is well documented in this animal model [11, 27, 28]. Altogether, the APP/PS1 mouse serves a proper subject for unveiling the molecular mechanisms linked to pathologically increased complement-driven synapse loss due to overproduction of toxic amyloid species. We addressed this question conducting systematic proteomic investigations on the C1q-tagged synaptic compartment of APP/PS1 mice to reveal the molecular mechanisms involved in neurodegeneration.

## Materials and methods

### Animals

The studies were carried out on 8–12 months of age and 24 months of age male APP/PS1 (B6C3-Tg(APPswe,PSEN1dE9)85Dbo) and C57BL/6N (wild-type) mice. Animals were housed under standard laboratory conditions (12:12 h light–dark cycle, with free access to water and food).

### Preparation of the fraction of synaptosomes

Animals were transcardially perfused with ice-cold phosphate-buffered saline under urethane anesthesia. Their brains were quickly removed, and the cerebral cortices from both hemispheres were isolated. The fraction of synaptosomes, comprising the pre- and postsynaptic compartments, was prepared immediately from cerebral cortices according to the protocol of Phillips et al. [29]. Briefly, isolated cortices were mechanically homogenized in an isoosmotic sucrose medium, and then samples were subjected to density-gradient centrifugation using two layers with different sucrose concentrations. The synaptosomes were collected from the interface and stored at 4 °C until use.

### Immunolabeling of synaptosomes

Freshly prepared synaptosomes were subjected to immunolabeling using our previously described protocol [20]. In brief, synaptosomes were gently fixed using 0.25% (wt/vol) formaldehyde in 320 mM sucrose, 1 mM EDTA, 5 mM Tris, pH 7.4 buffer (SET buffer). The low ionic strength of the SET buffer prevents synaptosome aggregation [20], and thus was used as the medium for the entire immunolabeling procedure. Lightly fixed synaptosomes were incubated with 1% (wt/vol) bovine serum albumin (BSA) to block aspecific antibody–antigen interactions. Subsequently, synaptosomes were incubated with anti-C1QA primary antibody (1:100 dilution; catalog number: ab155052; Abcam, Cambridge, UK), and then with Cy5-conjugated anti-rabbit secondary antibody (1:1,000 dilution; catalog number: 711-175-152; Jackson ImmunoResearch Laboratories, West Grove, PA, USA) after washing. Finally, samples were extensively washed, filtered through a 5.0 µm Durapore membrane filter (catalog number: SVLP02500; Merck Millipore, Billerica, MA, USA), supplemented with 0.5% (vol/vol) Pluronic F-68 detergent (Thermo Fisher Scientific; Waltham, MA, USA), and subjected to fluorescence-activated synaptosome sorting.

In the case of additional flow cytometry experiments, synaptosome suspensions were either labeled for mitochondrial markers or septin-3 (Sept3) after labeling for C1q, before synaptosome filtration. Samples assigned for detection of mitochondrial functions were labeled with the mitochondrial marker MitoTracker Green FM (catalog number: M7514; Thermo Fisher Scientific) and the mitochondrial superoxide indicator MitoSOX Red (catalog number: M36008; Thermo Fisher Scientific) at the same time, according to the manufacturer’s instructions. To assess synaptic Sept3 levels, another experimental group of synaptosome fractions was subjected to permeabilization using SET buffer containing 0.2% (vol/vol) Tween-20 and 1% (wt/vol) BSA. Then, synaptosomes were labeled first with anti-septin-3 primary (1:100 dilution; catalog number: MABN150; Merck Millipore), and then Alexa Fluor 488-conjugated anti-mouse secondary (1:1,000 dilution; catalog number: 715-545-151; Jackson ImmunoResearch Laboratories) antibodies and filtered.

### Flow cytometry and sorting of synaptosomes

The flow cytometry and sorting experiments were carried out using a BD FACSAria III cell sorter (BD Biosciences, San Jose, CA, USA) coupled with the BD FACSDiva software (BD Biosciences). Droplets were generated using a 70 µm nozzle, and synaptosomes were illuminated with a 488 nm- and a 633 nm-wavelength laser. Parameters for signal detection were set as follows: forward light scatter (FSC) detector photomultiplier tube (PMT) gain setting = 350 V with a 1.5 neutral density filter; side light scatter (SSC) detector PMT gain setting = 600 V; FSC threshold = 5,000; SSC threshold = 2,000; fluorescein isothiocyanate (FITC) channel PMT gain setting = 455 V; peridinin-chlorophyll cyanine 5.5 dye (PerCP-Cy5.5) channel PMT gain setting = 580 V, and allophycocyanin (APC) channel PMT gain setting = 508 V. Synaptosome suspension dilution and flow rate were adjusted to ensure optimal event recordings (below 20,000 processed events per sec). Gating of synaptosomes was performed using the fluorescence intensities of non-labeled synaptosome suspensions, i.e., that incubated with neither the mitochondria-specific dyes nor the primary antibodies but only with the corresponding secondary antibodies. For the sorting experiments, fluorescent C1q-tagged and non-fluorescent C1q-untagged synaptosomes were collected into separate tubes in “purity” sorting mode. Three million C1q-tagged and three million untagged synaptosomes were collected for the proteomics experiments from each animal. Sorted samples were concentrated with 100 K Amicon Ultra-15 centrifugal filter units (Merck Millipore), and proteins were precipitated with the chloroform–methanol precipitation method [30]. Precipitated proteins were resuspended in a lysis buffer (7 M urea, 2 M thiourea, 4% (wt/vol) CHAPS, 20 mM Tris, and 5 mM magnesium acetate, pH 8.5) suitable for the downstream proteomics studies and stored at − 80 °C.

In addition, quantitative flow cytometry measurements were conducted to evaluate the extent of colocalization between synaptic C1q and Sept3 proteins and the correlation between C1q and mitochondrial superoxide levels. Synaptically deposited C1q and synaptosomal Sept3 levels were assessed using the APC and FITC channels, respectively. In case of the mitochondrial study, we utilized the FITC, PerCP-Cy5.5, and APC channels for MitoTracker Green, MitoSOX Red, and C1q detection, respectively, using a parallel laser arrangement that ensured the necessary spectral separation of the fluorophores. In each experimental group, multiparametric data of 250,000 single synaptosomes were acquired and recorded for further analysis. Data processing and display were performed using FCS Express 6 software (De Novo Software, Glendale, CA, USA). Statistically significant differences between groups were determined with either two-tailed Student’s *t*-test of paired samples or one-way ANOVA followed by Tukey’s post hoc test. For analysis aiming at the evaluation of the correlation between synaptosomal C1q and Sept3 levels, we used the non-parametric Spearman’s rank correlation statistical test because neither of the data showed normal distribution.

### Proteomics investigation

The proteomic comparison of sorted synaptosomes was performed using the gel-based two-dimensional difference gel electrophoresis (2D-DIGE) proteomics technique. In study #1, comparisons were carried out between the C1q-tagged synaptosomes of six 8–9 months of age APP/PS1 and six age-matched wild-type (C57BL/6N) mice. For study #2, the proteomes of C1q-tagged and untagged synaptosomes were compared, prepared from six 8–9 months of age APP/PS1 mice. In both experiments, we performed the saturation labeling technique and protein separation steps, as described in details before [20]. In brief, synaptic proteins from the sorted samples were labeled with a highly sensitive cyanine-5 (Cy5) fluorescent dye and mixed with an internal control sample (pooled from the samples to be compared) that was labeled with the spectrally separable Cy3 dye. Protein mixtures then were subjected to protein separation, first, according to their isoelectric points, and then on the basis of their different molecular weights. Fluorescence of the separated proteins in the gel was recorded using a Typhoon TRIO + fluorescence scanner (GE Healthcare, Little Chalfont, UK). Protein spots were detected, matched, and their fluorescence intensities (normalized to that of the internal control sample) were compared between the different samples using the DeCyder 2D Differential Analysis Software (GE Healthcare). Statistically significant differences were determined in the software with two-tailed Student’s *t*-test of independent and paired samples in study #1 and study #2, respectively. The significance level was set at *P* < 0.05. Significantly altered protein spots that showed more than ± 1.1-fold change between the experimental groups were assigned for manual excision from a preparative gel containing 800 µg total synaptosomal proteins. Excised gel spots were placed in 1% (vol/vol) acetic acid solution and stored at 4 °C until protein identification.

For the mass spectrometric protein identification, proteins were digested with Trypsin Gold (Promega, Madison, WI, USA) according to the protocol by Shevchenko et al. [31] with minor modifications. The high-performance liquid chromatography–tandem mass spectrometry (HPLC–MS/MS) analysis was carried out employing a nanoflow UHPLC system (Dionex UltiMate 3000 RSLCnano System; Thermo Fisher Scientific) coupled to a high-resolution QTOF mass spectrometer (Maxis II ETD, Bruker Daltonik, Bremen, Germany). A CaptiveSpray source and a nanoBooster tank, filled with acetonitrile and operating with N_2_-flow at 0.2 bar, were fitted with the mass spectrometer. Desalting was conducted online on an Acclaim Pepmap C18 trap column (100 μm i.d. × 20 mm; Thermo Fisher Scientific), and then tryptic peptides were separated on a reverse-phase Acclaim Pepmap RSLC analytical column (C18, 75 μm i.d. × 150 mm; Thermo Fisher Scientific). Peptide elution from the analytical column to the emitter tip was performed using a flow rate of 300 nL/min and a 90 min-long gradient ranging from 2.5% to 45% (vol/vol) solvent B (solvent “A” was water containing 0.1% (vol/vol) formic acid, and solvent “B” was acetonitrile containing 0.1% (vol/vol) formic acid). Mass spectrometry utilized electrospray ionization in the positive-ion mode. Signal intensities were measured using single-stage mass spectrometry with settings as follows: capillary voltage = 1.2 kV, drying gas flow rate = 3 L/min, gas temperature = 150 °C. Protein identification employing tandem mass spectrometry used the AutoMS/MS setup (data-dependent analysis) from the *m/z* range of 300–2,200. Collision gas was N_2_, and the collision energy was varied in the 10–80 eV range according to predefined charge state-dependent configuration files. Protein content, identified from data-dependent analysis measurements, was searched against the latest SwissProt sequence database using the taxonomy of *Mus musculus* employing the Mascot Server v.2.5 (Matrix Science, London, UK). In the course of database searching, one missed cleavage was allowed, carbamidomethyl cysteine was set as a fixed modification, and methionine oxidation was allowed as variable modification. Identified proteins were accepted if a minimum of two unique peptides were accurately identified. For protein data analysis, we used ProteinScape v3.0 bioinformatics platform (Bruker, Billerica, MA, USA).

### Bioinformatics analysis

The biological function and subcellular localization of the identified proteins were evaluated using the Pathway Studio software (Elsevier Life Science Solutions), the Gene Ontology resource (GO; https://geneontology.org/), and the UniProt Knowledgebase (https://www.uniprot.org/). Enrichment analyses were conducted using the Pathway Studio software via the “Sub-Network Enrichment Analysis” tool utilizing databases of both the Pathway Studio and the GO knowledgebase. Accordingly, we addressed cell processes, protein and small molecule regulators, and diseases that are most probably linked to the analyzed protein datasets. The analysis is based on the calculation of a series of Fisher’s exact tests using 2 × 2 matrices. During each calculation, the matrix contains the number of altered proteins from our experimental data grouped as either related or unrelated to the particular entity (i.e*.*, cell process, protein and small molecule regulator, or disease) based on the exceptionally comprehensive knowledgebase of Pathway Studio. The matrix also involves the given entity’s total number of connections within the database and the total number of entities as well. The analysis provides probability values on how likely each entity in the entire database is linked to the experimental protein list. Statistically significantly enriched entities (*P* < 0.05) were ranked based on their calculated *P* values. When up- or downstream regulators of the altered proteins were evaluated, the direction of the connection between the protein and the given entity was also taken into account. We also carried out an additional enrichment analysis using the tool of the GO Consortium (https://geneontology.org/). The PANTHER overrepresentation analysis provided statistically significantly enriched GO terms within our experimental data using Fisher’s exact test with the conservative Bonferroni correction. Only GO terms with Bonferroni-corrected *P* values < 0.05 were considered as statistically significant.

### Immunofluorescence staining of mouse brain sections

The APP/PS1 and wild-type mice were anesthetized with urethane and transcardially perfused first with 0.1 M phosphate buffer (PB), pH 7.4, and then with 2% (wt/vol) formaldehyde in 0.1 M PB. Next, the brains were removed, post-fixed in the same fixative solution for an additional 3 h at room temperature (RT), and then washed and stored in 0.1 M PB containing 0.1% (wt/vol) sodium azide. Sixty-µm sagittal brain sections were produced with a vibratome and washed first with 0.1 M PB, and then Tris-buffered saline (TBS). Subsequently, brain sections were blocked in blocking buffer (100 mM L-lysine, 3% (wt/vol) BSA, 0.2% (vol/vol) Triton X-100 in TBS, pH 7.4) for 45 min at RT and incubated in primary antibody buffer (100 mM L-lysine, 1% (wt/vol) BSA in TBS, pH 7.4) with anti-C1q (1:1,000 dilution; catalog number: 182451; Abcam), anti-Syp (1:500 dilution; catalog number: 101 006; Synaptic Systems, Göttingen, Germany), and either anti-septin-3 (1:1,000 dilution; catalog number: MABN1530; Merck Millipore) or anti-septin-5 (1:500 dilution; catalog number: ab69538; Abcam) primary antibodies for 48 h at 4 °C. After several washing steps with TBS, the sections were incubated with Alexa Fluor 488-conjugated anti-mouse (1:400 dilution; catalog number: 715-545-151; Jackson ImmunoResearch Laboratories), Alexa Fluor 555-conjugated anti-rabbit (1:400 dilution; catalog number: A-31572; Thermo Fisher Scientific), and Cy5-conjugated anti-chicken (1:400 dilution; catalog number: 703-175-155; Jackson ImmunoResearch Laboratories) secondary antibodies in TBS for 3 h at RT. The brain sections were extensively washed with TBS, mounted on glass slides using AquaPoly/Mount medium (Polysciences, Warrington, PA, USA) and covered with cover glass (Marienfeld No. 1.5H, catalog number: 0107222).

### High-resolution confocal microscopy and image analysis

Immunolabeled brain sections were examined with the Leica HyVolution 2 pseudo-super-resolution imaging technique (Leica Microsystems, Wetzlar, Germany) using a Leica TCS SP8 stimulated emission depletion (STED) microscope equipped with a Leica HC PL APO 100 × STED white objective (numerical aperture = 1.4). The Alexa Fluor 488, Alexa Fluor 555, and Cy5 dyes were sequentially excited at 488 nm, 552 nm, and 638 nm, respectively, and fluorescence images were acquired between 500–540 nm, 560–600 nm, and 650–690 nm, respectively, employing spectral detections with a hybrid detector. Image restoration was conducted using the Huygens Pro deconvolution software, and Leica LAS X 3.1.1 software was utilized for image analysis.

The lateral and axial dimensions of the recorded images were ~ 20 × 20 µm, and ~ 5 µm, respectively. To evaluate the degree of colocalization between synaptic C1q and Sept3/5 proteins, we utilized an automated, Fiji image analysis platform-based [32] workflow combining a series of its plugins. First, high-resolution microscopy images were subjected to segmentation. Local maxima of individual synaptophysin (Syp), C1q, and Sept3/5 fluorescent spot-like objects were identified by the 3D Maxima Finder plugin, and then high-fidelity reconstruction of the spots was acquired using the “local mean” thresholding method in the 3D Spot Segmentation plugin. Geometrical centers (centroids) of the segmented Syp, C1q, and Sept3/5 spots were identified, and their pairwise colocalization analysis was performed using the JACoP (“Just Another Colocalization Plugin”) [33] via calculating the distances between centroids. The software automatically calculated an ellipsoid around every centroid with the maximal lateral and axial sizes of 200 nm and 500 nm, respectively (reflecting the point spread function of the object after image acquisition and processing). Colocalizing spots were accepted if their ellipsoids were overlapped. To evaluate the colocalization between exclusively synaptic C1q and Sept3/5 proteins, the positions of C1q and Sept3/5 spots that colocalized with Syp as well were compared. Statistically significant differences in the degree of colocalization between APP/PS1 and wild-type mice were determined with two-tailed Student’s *t*-test of independent samples. To group segmented Sept3/5 immunopositive spots based on their intensities, we obtained all the spot local maxima values (“peaks”) and calculated the mean pixel intensity and standard deviation for each image according to the pixel intensity frequency distribution. Subsequently, spots were assigned to any of the twelve intensity groups that were defined based on their peaks’ distance from the mean (*µ*) (± 0.25-, 0.5-, 0.75-, 1.0-, 1.25-, and 1.5-fold standard deviation (*σ*), excluding the artifact-prone spots with peaks either below µ − 1.5*σ* or above µ + 1.5*σ*). Correlation between the degree of colocalization and the corresponding intensity group was assessed by calculating Pearson’s correlation coefficient and two-tailed *P* value.

## Results

### Marked alteration of mitochondrial protein levels in C1q-tagged synapses of APP/PS1 mice

**Fig. 1:**
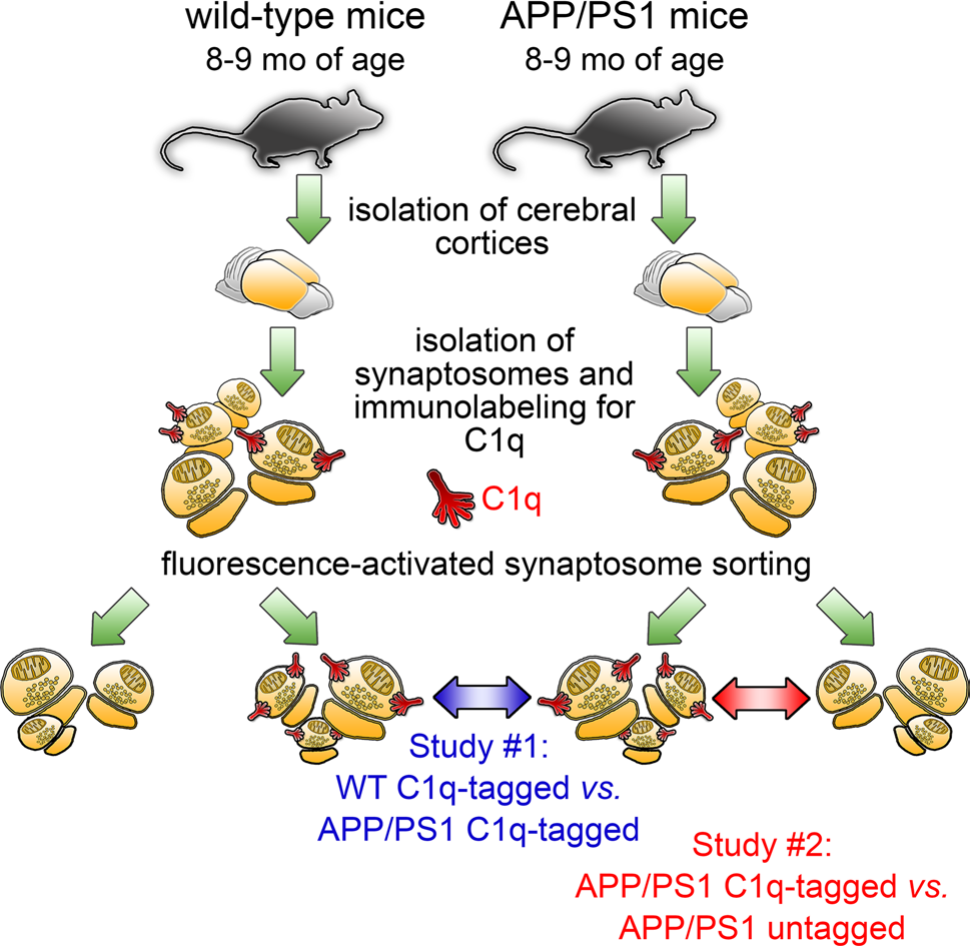
Experimental scheme of the high-throughput proteomic studies on sorted synaptosomes. Cerebral cortical synaptosomes, prepared from wild-type and APP/PS1 mice, were immunolabeled for C1q, and then inherently C1q-tagged and untagged synaptosomes were separated using fluorescence-activated synaptosome sorting. In study #1, proteomic comparison of C1q-tagged synaptosomes was conducted between wild-type and APP/PS1 mice. Study #2 was designed to identify protein-level differences between C1q-tagged and untagged synaptosomes prepared from APP/PS1 mice

**Fig. 2:**
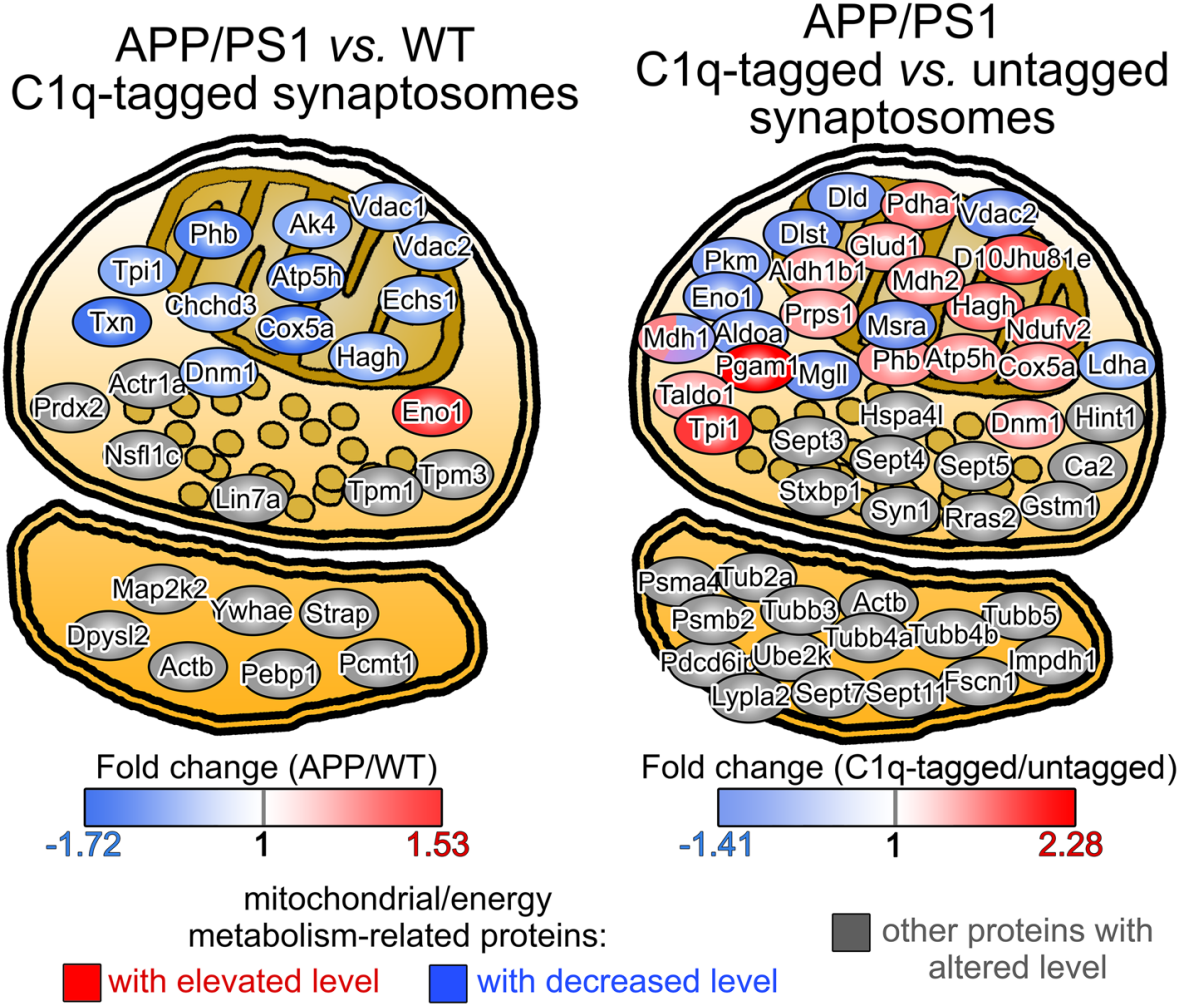
Mitochondrial and energy metabolism-related proteins identified in the two studies. On the schematic illustration of synaptosomes comprising a presynaptic compartment with mitochondrion and a pool of synaptic vesicles oppositely to the postsynaptic part, the entire list of altered proteins is presented as ellipses. Proteins are placed according to their known subcellular locations in case of the mitochondrial and synaptic vesicle-related ones, while other proteins were distributed throughout the pre- or postsynaptic cytoplasm. The illustration focuses on the wide array of mitochondrial and energy metabolism-related proteins identified in both studies and colored based on the degree of their level changes

Complement-mediated synaptic pruning occurs under both physiological and pathological conditions. In our previous study, we isolated and analyzed the proteome of C1q-tagged and untagged synaptosomes prepared from the cerebral cortices of healthy, adult, wild-type mice to characterize those synapses that are predisposed to complement-dependent microglial elimination [20]. However, it is still unclear whether normal and aberrant synapse loss due to AD-related neurodegeneration can be distinguished based on their different synaptic functional disturbances. Thus, our current objective was to address whether C1q-tagged synapses exhibit robust differences in their proteome between wild-type and APP/PS1 mice (study #1; the experimental scheme is shown in Fig. 1). We purified synaptosome fractions from the cerebral cortices and selectively sorted immunolabeled C1q-tagged synaptosomes taking advantage of our thoroughly validated and recently published fluorescence-activated synaptosome sorting protocol [20]. Proteomic comparison of sorted synaptosomes’ lysates was performed with the 2D-DIGE method, and altered proteins were identified using mass spectrometry. The investigation revealed statistically significant alterations of 15 protein spots above the predefined criteria, which comprised 26 synaptic proteins (Table S1). Interestingly, we observed a pronounced and general decrease in the level of the changed proteins in the APP/PS1 mice, as 12 out of the 15 protein spots showed lower fluorescence intensity levels in comparison with the wild-type mice. The proteins’ classification based on their cellular functions and subcellular locations unveiled that an outstanding portion of the identified proteins is linked to energy metabolism and the mitochondrial compartment (13 out of 26 proteins; Fig. 2).

Enrichment analysis suggested that the most probable cell process regulated by the identified proteins is apoptosis (Fig. S1). Amyloid precursor protein and hydrogen peroxide (H_2_O_2_) were identified as the most likely protein and small molecule regulator candidates, respectively; while, AD was ranked first based on its probability score among the diseases potentially linked to the dataset (Fig. S1). Collectively, enrichment analysis results reflected the AD-related molecular phenotype in the differentially regulated proteins and raised the possible impairment of the redox homeostasis within the synapse. Moreover, the PANTHER overrepresentation test, employing a conservative statistical approach, concluded the sole enrichment of the GO term “pyruvate metabolic process” within the altered proteins. Altogether, the results support the idea that toxic Aβ accumulation leads to disturbances in mitochondrial functions that can amplify synaptic C1q deposition with the possible involvement of apoptotic mechanisms and oxidative stress.

### Mitochondrial oxidative stress is linked to the accumulation of C1q on the synaptic surface

We also carried out the protein-level comparison of C1q-tagged and untagged cortical synaptosomes obtained from APP/PS1 mice (study #2; Fig. 1) in the same manner as described in study #1. According to the results of the analysis, 46 protein spots showed altered levels, which were matched to 50 proteins identified using mass spectrometry (Table S2). In contrast to the previous experiment, the directions of level changes were more balanced, as we detected 17 decreased and 29 increased protein spots in the C1q-tagged *vs.* untagged synaptosome comparison. Again, the differentially regulated proteins were enriched in those that are coupled to energy metabolism, localized in the mitochondria or both (25 out of the 50 proteins; Fig. 2). Enrichment analysis also highlighted apoptotic processes, in accordance with our prior data [20] (Fig. S2). Glucose was identified as the most probable small molecule regulator candidate, which was followed again by H_2_O_2_ (Fig. S2). Proteins were most likely enriched in those related to schizophrenia, while AD was ranked second (Fig. S2). Interestingly, RAC-alpha serine/threonine-protein kinase (Akt1) appears as the protein candidate to regulate the altered proteins with the highest probability (Fig. S2). According to a recent study, reactive oxygen species (ROS) detrimentally affect synaptic Akt1-signaling that eventually contributes to synaptic dysfunction in AD pathogenesis [34]. In addition, the PANTHER overrepresentation analysis revealed the enrichment of the GO term “canonical glycolysis” along with many of its “child” terms, e.g., the “pyruvate metabolic process”.

**Fig. 3:**
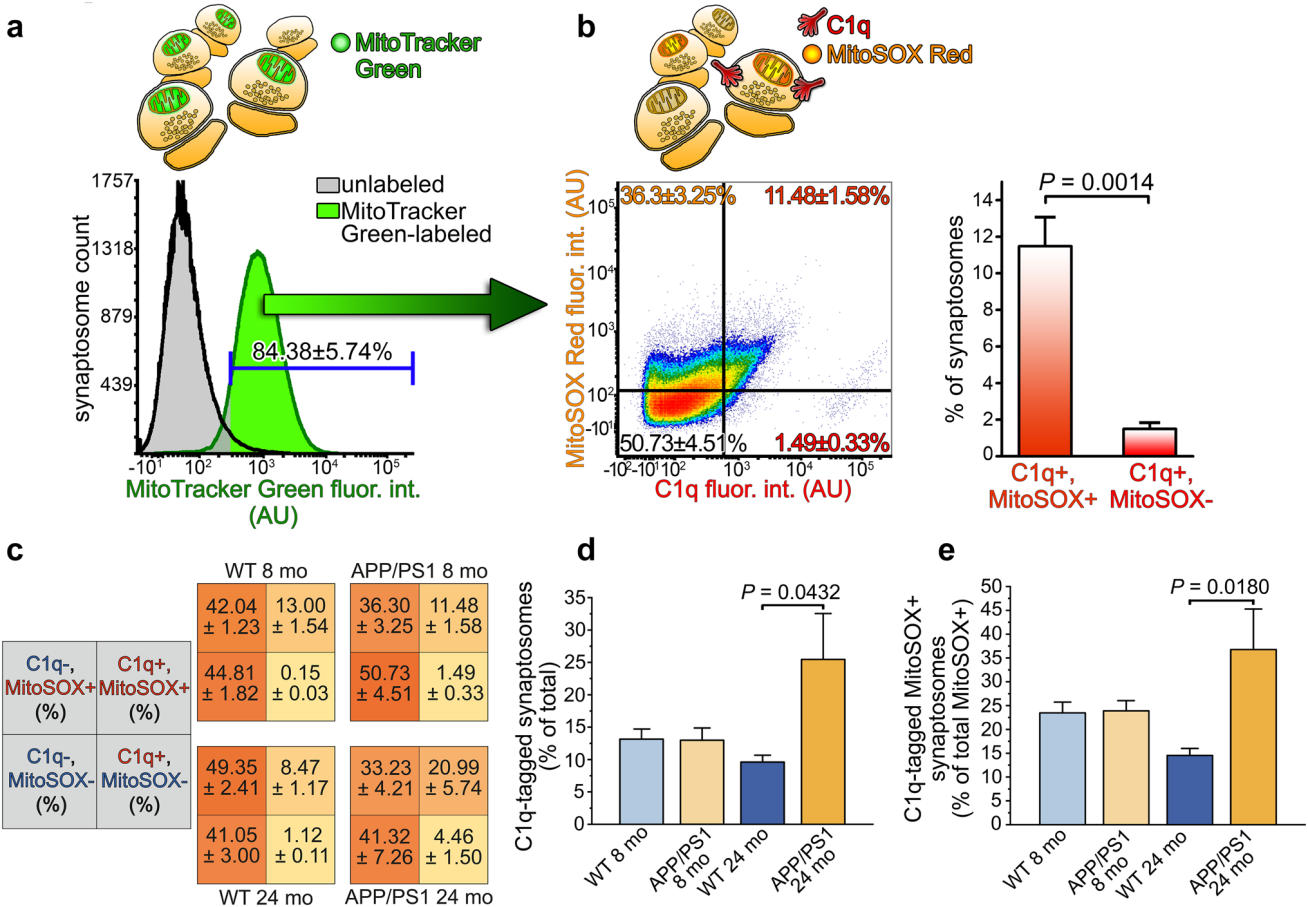
C1q-tagged synaptosomes of APP/PS1 mice are characterized by elevated mitochondrial superoxide levels. **a** Representative overlaid histograms show the results of the flow cytometry analysis of APP/PS1 mice synaptosomes labeled with the mitochondrial marker MitoTracker Green. Based on the gating criterion set according to the fluorescence intensity of the unlabeled sample, 84.38 ± 5.74% of the synaptosomes contained mitochondria. **b** Representative density plot of APP/PS1 mouse synaptosomes with mitochondrial content demonstrating that the vast majority of C1q-tagged synaptosomes possesses mitochondria with excessive superoxide levels. The plot is based on the labeling results for C1q and the mitochondrial superoxide indicator MitoSOX Red and gating parameters were set according to the unlabeled samples. The bar graph shows the statistically significant difference between C1q and MitoSOX Red double-labeled and C1q-tagged but MitoSOX Red-negative synaptosome amounts. **c** Additional summarized flow cytometry results involving synaptosome-labeling data of 8 and 24 months of age wild-type and APP/PS1 mice. Each quadrant shows the percentage of the labeled population, as indicated in the gray quadrants on the left. **d**,** e** The bar graphs show the statistically significant differences in the percentage of C1q-tagged synaptosomes between animal groups [*F* (3, 13) = 3.55, *P* = 0.0451] and in the percentage of MitoSOX Red and C1q double-labeled synaptosomes among the MitoSOX Red-positive ones [*F* (3, 13) = 414.93, *P* = 0.0288], respectively. Means ± S.E.M. are shown. *n* = 4–5 mice per animal group. Two-tailed Student’s *t-*test of paired samples (**b**) and one-way ANOVA followed by Tukey’s post hoc test (**d** and **e**) were used for the comparisons

Regarding mitochondrial disturbances, functional impairments in mitochondria favor the local overproduction of ROS, the by-products of ATP generation, and hence, substantially hamper normal neuronal functions [35]. Mitochondrial ROS production is responsible for neuronal damage to a large extent because the mitochondrion is a major source of ROS in neurons, which has an outstandingly high ATP demand [36]. We investigated the correlation between synaptically elevated mitochondrial ROS level and C1q-tagging to find the connection between mitochondrial dysfunction and predisposition to synaptic complement deposition. Cortical synaptosomes from APP/PS1 mice were immunolabeled for C1q and labeled with both mitochondria-specific and mitochondrial ROS-specific dyes. Synaptosome suspensions were subjected to multiparametric flow cytometry analysis. After setting the gate based on the negative control sample, only those synaptosomes were analyzed further that contained mitochondria (84.38 ± 5.74% of synaptosomes recorded, mean ± S.E.M.; Fig. 3a). Our flow cytometry results revealed that the C1q-tagged subpopulation of synaptosomes was almost entirely positive for the mitochondrial ROS-specific dye, MitoSOX Red, which becomes fluorescent upon oxidation by mitochondrial superoxide (SOX) (Fig. 3b). On the other hand, mitochondrially accumulated SOX alone does not necessarily attract synaptic C1q deposition, as suggested by the high amount of C1q-untagged synaptosomes with high SOX level (Fig. 3b). It has to be noted that additional ROS production could occur during isolation of synaptosomes that might cause more MitoSOX Red-positivity compared to that of the in vivo state but likely independent of C1q deposition. The same flow cytometry experiments were also carried out on wild-type mice, showing that C1q-tagged synaptosomes are primarily MitoSOX Red-positive (Fig. 3c), thus suggesting that the C1q-tagged synapses are characterized by mitochondrial superoxide anion accumulation both in the wild-type and the Alzheimer’s disease model mouse strains. Notably, neither the expected increase in the percentage of C1q-tagged synaptosomes in the APP/PS1 mice nor any alteration in the amount of MitoSOX Red-positive synaptosomes was detectable employing 8 months of age mice with this technique (Fig. 3c). We extended the flow cytometry experiments to 24 months old APP/PS1 and wild-type mice to gain insight into the well-known age-dependent and Aβ accumulation-related [11] increase of synaptic C1q-tagging and to investigate its hypothesized connection with impaired mitochondrial functions. Our additional experiments again demonstrated the preferential accumulation of C1q on synapses with compromised mitochondrial functions in both mouse strains (Fig. 3c). Moreover, we observed a statistically significantly higher accumulation of C1q onto synapses in aged APP/PS1 mice in comparison with the aged wild-type ones suggesting overactivation of the complement within the cerebral cortex (Fig. 3d). The enhanced synaptic C1q-deposition was accompanied by a statistically significant elevation in the number of MitoSOX Red- and C1q double-positive synaptosomes among the MitoSOX Red-positive ones in comparison with the wild-type mice, strongly implying that increased C1q-accumulation in severe neurodegeneration targets synapses with dysfunctional mitochondria (Fig. 3e). To summarize the flow cytometry results, high mitochondrial superoxide level in the C1q-tagged synapses is presumably a general and mouse strain-independent phenomenon. Importantly, age-dependent excessive C1q binding to the synapse in the APP/PS1 mice primarily targets synapses possessing dysfunctional mitochondria.

Together, the great extent of mitochondrial protein-level changes and the detected oxidative stress condition in almost all of the C1q-tagged synaptosomes strongly supports the idea of mitochondrial functional imbalance in the background of synaptic C1q accumulation in APP/PS1 mice.

### Accumulation of septin proteins in the C1q-tagged synapses of APP/PS1 mice

**Fig. 4:**
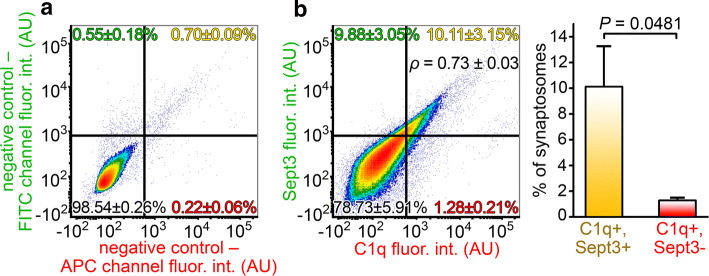
Accumulation of Sept3 in the C1q-tagged synaptosomes. **a** Representative flow cytometry density plot showing the fluorescence intensity of solely secondary antibody-labeled, negative control synaptosomes on the relevant fluorescence channels. **b** According to the gating criteria, the fluorescence of synaptosomes simultaneously labeled for C1q and Sept3 was evaluated. A strong positive correlation is apparent between synaptic C1q and Sept3 levels (analysis of synaptosomes in the top right quadrant: *ρ* = 0.73 ± 0.03, *P* < 0.0001 for each sample), and synaptic C1q shows remarkable colocalization with Sept3 on individual synaptosomes. The bar graph on the right-hand side demonstrates the statistically significant difference between C1q and Sept3 double-labeled and Sept3-lacking C1q-tagged synaptosomes. Means ± S.E.M. are shown. *n* = 5 APP/PS1 mice. For each biologically independent sample, Spearman’s rank correlation analysis was used for assessing the correlation between C1q and Sept3 levels, while statistically significant correlations were identified by determining the corresponding two-tailed *P* values. Two-tailed Student’s *t-*test of paired samples was used for comparison between quadrants

Further evaluation of the proteomic dataset obtained from the comparison of C1q-tagged and untagged synaptosomes from APP/PS1 mice pointed out the strikingly high enrichment of septin proteins (identification of Sept3, Sept4, Sept5, Sept7, and Sept11; Table S2). Septins are known as multifunctional intracellular GTPases capable of forming filamentous and ring-like hetero-oligomeric structures [37]. In the presynaptic compartment, septins (e.g., Sept3, Sept4, and Sept5) are involved in the regulation of synaptic vesicle exocytosis and recycle [38–40]. Postsynaptically, certain septin family members (e.g., Sept7 and Sept11) form a protein assembly that serves as a physical barrier close to the bases of dendritic spines [41, 42]. Due to their fundamental synaptic roles and marked alteration in our study, we further sought to investigate septins. Among them, the presynaptically enriched Sept3 [43] was assigned for the examinations first because it showed an extensive and exceptionally high elevation in its levels in the C1q-tagged synaptosomes compared to the untagged ones: the increased Sept3 abundance was detected in four protein spots with a 3.24-fold increase in one of them (Table S2). Synaptosome fractions from APP/PS1 mice were double-labeled for C1q and Sept3, and their co-occurrence was quantitatively assessed using flow cytometry. The resulting data showed a remarkably strong positive correlation between synaptosomal C1q and Sept3 levels (Spearman’s rank correlation coefficient of samples  *p*= 0.73 ± 0.03, mean ± S.E.M.; two-tailed *P* < 0.0001 for each sample; Fig. 4). This result implies that synaptic Sept3 accumulation facilitates extracellular C1q deposition onto the synapse.

**Fig. 5:**
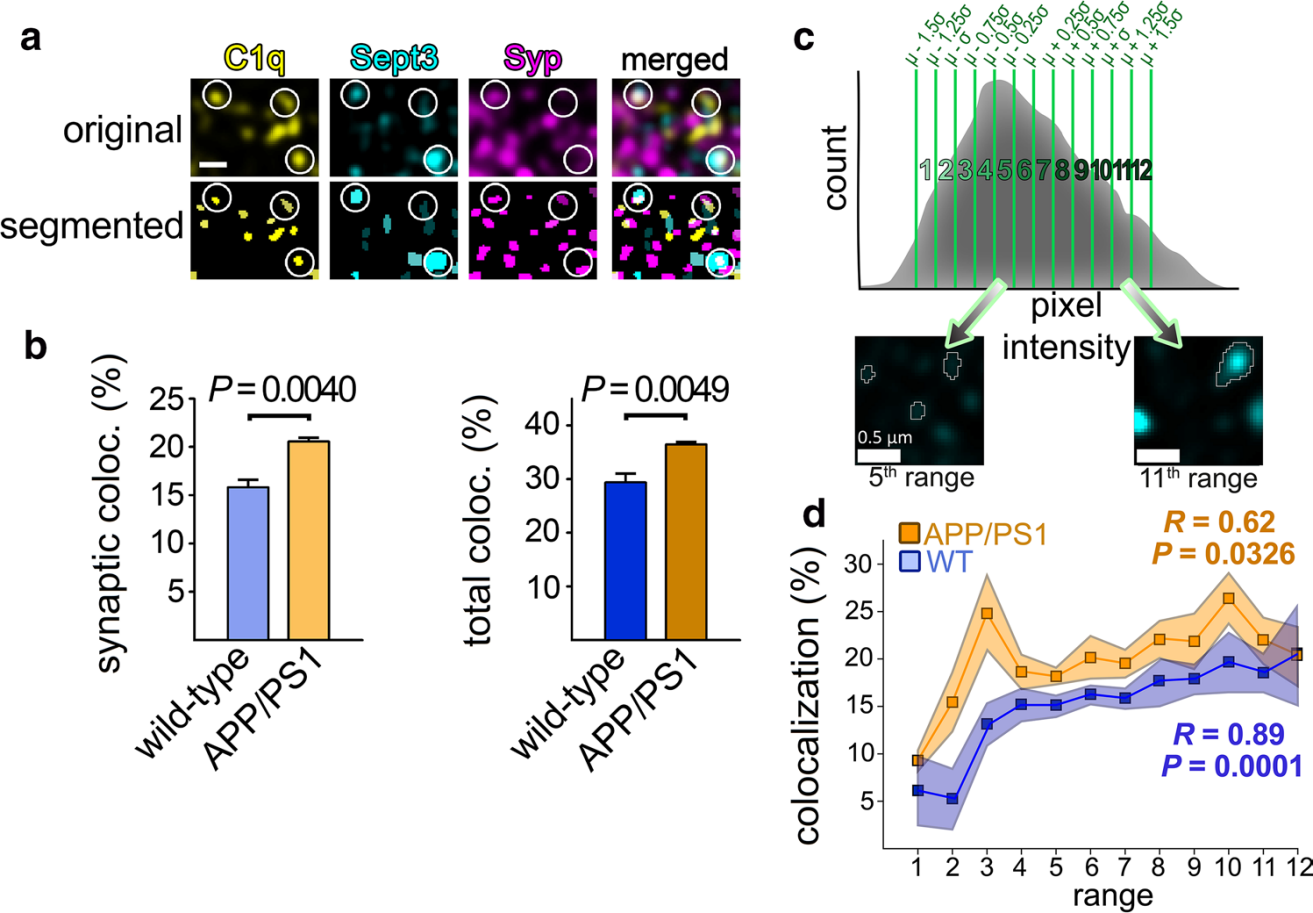
C1q accumulation in APP/PS1 mice is linked to Sept3 levels as demonstrated by immunostaining. **a** Representative high-resolution confocal microscopy image showing C1q, Sept3, and the synaptic marker Syp labeling in the cortical neuropil. To enable automatic colocalization analyses, labeling pattern was reconstructed for each protein. White circles indicate positions of colocalizations between the three proteins. **b** Bar graphs showing a statistically significant elevation in the percentage of synaptic and total Sept3 that colocalize with C1q in APP/PS1 mice compared to the wild-type ones. **c** Subdivision of Sept3 spots according to their fluorescence intensities as illustrated on a histogram showing spot counts as a function of the pixel intensity value of each spot’s local maximum. The mean pixel intensity (*µ*) and the standard deviation (*σ*) were determined for each image, groups were created, and every Sept3 spot was assigned to any of the groups based on its local maximum’s position on the pixel intensity range as depicted on the microscope images below. **d** Line graph demonstrating the correlation between the percentage of synaptic Sept3 colocalized with C1q and Sept3 levels based on the twelve predefined signal intensity ranges. A statistically significant positive correlation was revealed in both APP/PS1 and wild-type mice. Scale bar is 0.5 µm. Means ± S.E.M. are shown. *n* = 4–6 images from 2–3 animals per mouse strain. Two-tailed Student’s *t*-test of independent samples was used for the comparisons. Pearson’s correlation coefficient (*R*) and the two-tailed *P* value were determined for the correlation analyses

To investigate this phenomenon with a complementary approach, we carried out immunolabeling and high-resolution confocal microscopy of cerebral cortical sections of APP/PS1 and wild-type mice. First, we evaluated the extent of colocalization between synaptic C1q and Sept3 for both mouse strains. Although Sept3 is assumed to be a core component of the presynapse, its analysis was limited to those with a considerable amount in close proximity of Syp “spots” (Fig. 5a). We observed a higher level of synaptic Sept3 colocalizing with synaptic C1q in APP/PS1 mice compared to that in wild-type mice (1.30 ± 0.08-fold increase, mean ± S.E.M.; *P* = 0.0040; Fig. 5b). Similarly, elevated colocalization of total Sept3 and C1q proteins was seen in APP/PS1 mice, regardless of their synaptic or non-synaptic localization (1.24 ± 0.07-fold increase, mean ± S.E.M.; *P* = 0.0049; Fig. 5b). These latter data propose that overproduced C1q preferentially accumulates in regions with high Sept3 content even on other neuronal cell compartments besides the synapse (e.g., axons or cell soma). An increased amount of C1q-tagged synapses was also apparent in the AD mouse model compared to the wild-type mice (1.50 ± 0.24-fold increase, mean ± S.E.M.; *P* = 0.0321), in accordance with prior data [11]. Additionally, we tested whether the elevated synaptic Sept3 levels are linked to more abundant C1q deposition, as revealed by the flow cytometry data. To this end, synaptic Sept3 immunopositive spots were subdivided into twelve different groups ranging from the faintest to the brightest ones (Fig. 5c). Subsequently, the extent of colocalization with synaptic C1q was assessed for Sept3 group-wise. Supporting our flow cytometry results, we observed a moderately strong positive correlation between Sept3 levels and the degree of colocalization with C1q in APP/PS1 mice (Pearson’s correlation coefficient of means, *R* = 0.62; two-tailed *P* = 0.0326; Fig. 5d). In other words, synapses with higher Sept3 content are more likely to possess C1q than those with lower Sept3 levels. Similarly, this positive correlation was also apparent in the case of wild-type mice to an even larger extent (*R* = 0.89, two-tailed *P* = 0.0001; Fig. 5d).

To investigate further the presynaptic machinery Sept3 takes part in, we conducted additional examinations focusing on Sept5, which also showed elevated abundance in the C1q-tagged synaptosomes compared to the untagged ones in APP/PS1 mice (Table S2). In line with our results on Sept3, we observed enhanced colocalization of synaptic and total Sept5 proteins with C1q (1.19 ± 0.11-fold increase, *P* = 0.0444 and 1.61 ± 0.31-fold increase, *P* = 0.0402, respectively; means ± S.E.M.; Fig. S3a). However, contrasting with Sept3, no correlation was detected in synaptic Sept5 level and the degree of colocalization with C1q (Fig. S3b).

Taken together, the enhanced synaptic C1q deposition simultaneously with the elevation in the percentage of C1q-colocalizing synaptic Sept3 and Sept5 highly suggest that synaptic C1q accumulation in APP/PS1 mice occurs preferentially on synapses with considerable amounts of certain septins.

## Discussion

Excessive synapse loss is a hallmark of AD, which develops early in disease progression and profoundly linked to compromised cognitive functions. The robust appearance of gradually aggravating synapse elimination even enables it to be employed as a biomarker of the disease using a noninvasive imaging technique [44]. Consequently, uncovering background mechanisms that pose an increased risk for elevated, widespread synaptic dysfunction and ensuing synapse removal is of great importance to design efficient therapeutic strategies aiming the preservation of normal cognitive functions. Increased accumulation of cytotoxic Aβ aggregates in brain parenchyma due to elevated production and inefficient clearance is widely considered as a direct cause of synaptic failure [45]. Synaptotoxicity of amyloidogenic products is mainly attributed to the oligomeric form of Aβ (reviewed in [46]), and it was demonstrated that the amount of synaptically localized oligomeric Aβ positively correlates with excitatory synapse loss [47]. According to a growing body of data, damaged synapses became predisposed to engulfment by surrounding cells with phagocytic activity (mostly by microglia), which is even more pronounced as neuroinflammation arises due to the critical accumulation of amyloid plaques and neurofibrillary tangles [6]. The complement system recently emerged as one of the essential tools for surveilling microglia to aid the recognition of eliminable synapses. Analogous to their defensive roles peripherally, brain-derived complement components tend to deposit onto toxic amyloid plaques and neurofibrillary tangles [48]. In addition, upregulated local complement components also target synapses and directly cause pathological synaptic pruning. The assumed hypothesis on their role in enhanced synapse elimination is cemented by studies demonstrating protection against overabundant synapse loss via selective blocking of complement C1q [11] and genetic deletion of either C1q [11, 49], complement C3 [11, 27], or C3 receptor [11].

In our current study, we aimed to interrogate functional disturbances that predispose to Aβ-induced and complement-dependent microglial synapse removal. For this, we employed our recently optimized fluorescence-activated sorting procedure of cerebral cortical synaptosomes [20]. This workflow allows the reliable quantification of minute amounts of synaptic proteins, which is crucial due to the low protein yield of sorted C1q-tagged synaptosomes prepared from mice. Although the utilized 2D-DIGE gel-based proteomic technique provides excellent sensitivity for identifying even slight alterations in protein levels from low sample amounts [50], the coverage of the observable synaptic proteome is not complete. This limitation is rooted in the well-known obstacles of traditional 2D gel electrophoresis that prevent the routine detection of particular protein groups, e.g., hydrophobic membrane proteins and low-abundant ones [51]. Thus, false-negative proteins might have to be taken into consideration using this high-throughput approach, and proper validation experiments are necessary.

Our first study, addressing the molecular differences between C1q-tagged synaptosomes of wild-type and APP/PS1 mice, raised the general impairment of mitochondrial functions in the AD animal model. Additionally, enrichment of mitochondria-localized proteins was also apparent in the second study examining proteomic differences between C1q-tagged synaptosomes of APP/PS1 mice compared to untagged ones. Functional disturbances of the mitochondria, caused by, e.g., damage to the mitochondrial DNA and age-related functional decline can ultimately lead to increased mitochondrial ROS production that damages mitochondrial macromolecules and conveys elevated vulnerability for further adverse effects [52, 53]. Thus, the detection of mitochondrial ROS can provide insight into the general functional state of the mitochondrion. Mitochondria in C1q-tagged synaptosomes unambiguously showed elevated ROS levels, supporting the prevailing hypothesis that Aβ accumulation impairs synaptic mitochondrial functions. Amyloid precursor protein is capable to be translocated to the mitochondria [54] and can give rise to the deleterious over-production of Aβ locally, which targets many essential mitochondrial proteins (reviewed in [55]) and also induces extensive alterations in the mitochondrial proteome [25]. The pivotal role of mitochondria-related disturbances in AD is also supported by additional data demonstrating an outstanding enrichment of key AD-associated proteins, APP and presenilins, in the mitochondria-associated endoplasmic reticulum membrane [56]. This cell compartment exhibits early AD-linked proteomic changes in APP/PS1 mice [57]. It has been shown that the Aβ-elicited extensive synaptic failure leads to the manifestation of apoptosis-related processes in the synapse [58] and first increases synaptic C1q, and then C3 deposition [11]. “Eat-me” signals for complement components, such as externalized phosphatidylserine [59], could mark apoptotic synapses for C1q deposition [20]. However, the specific apoptotic pathway that triggers the apoptotic molecular cascade in the synapse prior to complement deposition remains unknown. According to the presented results, we suggest that the intrinsic, mitochondrion-dependent apoptotic cascade could markedly contribute to the Aβ-induced complement-mediated synapse loss. Nevertheless, future studies are required to clarify the exact role of the intrinsic pathway and to address whether the extrinsic arm of apoptosis is involved as well. In sum, our novel data links the mitochondrial theory of AD to the previously reported aggravating complement-dependent synapse loss characteristic for AD.

Besides mitochondrial disturbances, our proteomics and subsequent validation experiments clearly pointed to the involvement of the septin protein family in synaptic C1q deposition in AD-related pathology. Sept3 and Sept5 were not identified in study #1 but in study #2, and a higher degree of colocalization between synaptic Sept3/5 and C1q was described in the APP/PS1 mice compared to the wild-type ones. These results altogether suggest that although C1q-tagged synapses in the investigated mouse strains exhibit no overall major differences in their Sept3/5 levels, Sept3 and Sept5 accumulation is connected to a larger extent to synaptic C1q deposition in the AD animal model compared to the healthy mice. Interestingly, certain septins show accumulation at neurofibrillary tangles [60], and differential expression of septins have been observed in neocortical protein lysates of AD patients compared to controls [61]. Septins are fundamentally engaged in substantial synaptic functions. Presynaptic septins tightly regulate synaptic vesicle trafficking and exocytosis; moreover, neurite growth and maintenance (reviewed in [39]). Some of their postsynaptic counterparts influence formation of functional dendritic protrusions (Sept6 [62], Sept7 [63, 64], and Sept11 [41]), and Sept7 is reported to effectively suppress the lateral movement of membrane proteins by forming a barrier at the base of the dendritic spine [42]. We identified increased levels of Sept3, Sept5, Sept7, and Sept11 and decreased abundance of Sept4 in C1q-tagged synapses in comparison with the untagged ones in APP/PS1 mice (Table S2). Among them, we demonstrated the C1q-linked accumulation of Sept3 in the synapses and the enhanced colocalization of both Sept3 and Sept5 with C1q in the AD animal model in comparison to the wild-type mice. Although the absence of Sept3 and Sept5 does not affect negatively the proper synaptic transmission, possibly due to functional redundancy among septin proteins [65], their overabundant presence could be detrimental and eventually attract C1q. In agreement, experimental data demonstrated that Sept5 is an inhibitor of vesicle exocytosis [66] and prevents docking and exocytosis of synaptic vesicles by forming a filamentous barrier in immature synapses [67]. Accordingly, forchlorfenuron treatment, which specifically inhibits disassembly of the otherwise dynamic septin filaments leading to abnormally large septin polymers and clusters, potently decreases presynaptic neurotransmitter secretion [40]. Interestingly, overexpression of Sept5 causes neurodegeneration and a phenotype of parkinsonism by occluding dopamine release [68] and inducing cell death [69]. The potential synaptic effect of elevated Sept3 level, however, is still obscure. On the other hand, polymorphism of *Sept3* gene is associated with susceptibility to AD [70]. In addition, overexpressed Sept3 was demonstrated to form a filamentous structure in complex with Sept5 and Sept7 [71]. Taken together, these data raise the possibility that the build-up of presynaptic complexes comprising Sept3 and Sept5 might interfere with synaptic vesicle exocytosis, thus compromising synaptic transmission. Considering that low-activity synaptic contacts are predisposed to complement-mediated pruning [72], septin accumulation might convey elevated vulnerability to synaptic pruning by weakening the synapses.

The observed higher abundance of two constituents of the dendritic barrier, namely Sept7 and Sept11 in C1q-tagged synaptosomes suggests that molecular barriers might separate eliminable synapses from other parts of the dendritic branch. Taking into account that complement-tagged synapses show the molecular signatures of apoptotic-like processes [20], it can be hypothesized that this diffusion barrier might help to isolate and localize apoptotic compartments from other areas of the arborized neurites and the cell body. Spatial restriction of the apoptotic cascade’s propagation is crucial in maintaining the segregation of functional and dysfunctional areas of the cell. It has already been unveiled that the proteasomes critically contribute to preventing the erroneous reverse spread of apoptotic signaling from the synapse to the cell body [73]. Based on our results, septins could emerge as further players in this isolation process. Alternatively, it can also be hypothesized that the causal relationship’s direction is the opposite, and exaggerated accumulation of dendritic septins leads to synaptic dysfunction via obstructing membrane protein trafficking required to ensure synaptic plasticity. Finally, future studies will be needed to examine in detail alterations in synaptic Sept4 levels in relation to neurodegeneration-associated synapse loss. Intriguingly, one of its isoforms, the mitochondrion-localized ARTS, is known to effectively regulate apoptosis and translocate to the nucleus due to pro-apoptotic stimuli [74].

The direct effect of Aβ and the mechanistic steps that eventually lead to septin accumulation remain elusive. Similarly, the connection between mitochondrial processes and the organization of the synaptic septins also has to be clarified. However, previous studies demonstrated the capability of mitochondria to organize septin polymer formation [75] and the potential of septins to regulate mitochondrial fission [76] in non-neuronal cells.

**Fig. 6:**
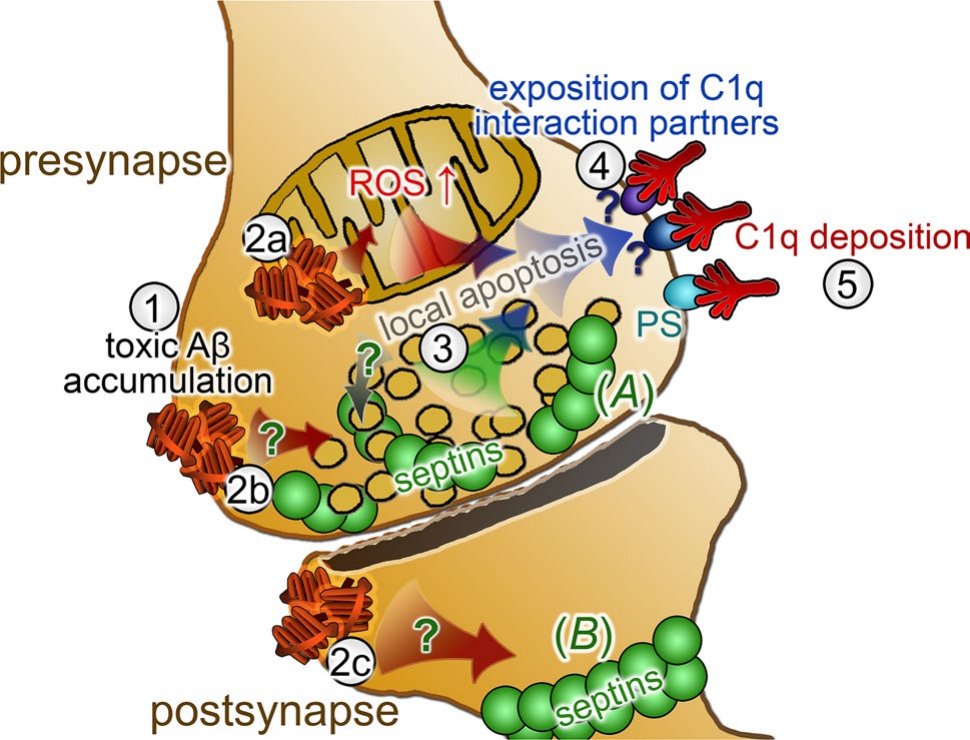
Overview of synaptic mechanisms linked to Aβ-induced C1q deposition based on the current study. According to the proposed model, the accumulation of Aβ (1) causes synaptic failure via different pathways leading to concomitant C1q deposition. Mitochondrial Aβ generation leads to mitochondrial dysfunction with excessive ROS production (2a), while Aβ targeting the pre- and postsynaptic regions (2b and 2c, respectively) could alter septin organization in both synaptic parts via mechanistically yet unknown processes. Presynaptic septins (e.g., Sept3 and Sept5) in the active zone show aberrant elevation linked to Aβ accumulation (*A*), while overabundant postsynaptic septins (e.g., Sept7, Sept11) might influence the organization of the dendritic septin barrier (*B*). Deteriorated mitochondrial functions along with compromised synaptic transmission due to presynaptic septin accumulation might converge into the activation of the already described local apoptotic-like processes (3). Alternatively, apoptotic mechanisms could also influence the formation of septin assemblies. Through these mechanisms, well-known [e.g*.*, phosphatidylserine (PS)] and so far hidden C1q interaction partners become exposed on the synaptic surface (4) that eventually lead to deposition of C1q (5)

In sum, our current results shed light on and emphasize the importance of the imbalance in mitochondrial functions and the organization of septins within the synaptic compartment in AD-related, complement-mediated synapse loss. We propose a model showing neurotoxic Aβ accumulation-induced mitochondrial impairments and septin-related changes that can result in the already revealed activation of local apoptotic-like mechanisms and subsequent synaptic C1q deposition (Fig. 6).

### Electronic supplementary material

Below is the link to the electronic supplementary material.

Supplementary file1 (PDF 921 kb)

## Abbreviations

2D-DIGE: Two-dimensional difference gel electrophoresis
AD: Alzheimer’s disease
APC: Allophycocyanin
APP: Amyloid precursor protein
Aβ: Amyloid-beta
BSA: Bovine serum albumin
Cy: Cyanine
FITC: Fluorescein isothiocyanate
FSC: Forward light scatter
GO: Gene ontology
PB: Phosphate buffer
PerCP-Cy5.5: Peridinin-chlorophyll cyanine 5.5
PMT: Photomultiplier tube
PS1: Presenilin-1
ROS: Reactive oxygen species
Sept: Septin
SET: Sucrose/EDTA/Tris
SOX: Superoxide
SSC: Side light scatter
Syp: Synaptophysin
TBS: Tris-buffered saline
