# Supplementary material for: Synaptic mitochondrial dysfunction and septin accumulation are linked to complement-mediated synapse loss in an Alzheimer’s disease animal model
Source: Cell Mol Life Sci. 2020 Feb 7;77(24):5243–58. doi: 10.1007/s00018-020-03468-0 (PMC7671981; doi:10.1007/s00018-020-03468-0)
Supplement: Supplementary file 1 — Supplementary file1 (PDF 921 kb) [file 18_2020_3468_MOESM1_ESM.pdf]

Supplementary material

Synaptic mitochondrial dysfunction and septin accumulation are linked to complement-mediated synapse loss in an Alzheimer's disease animal model

Balázs A. Györffy[1,2], Vilmos Tóth[2,3], György Török[4,5], Péter Gulyássy[6], Réka Á. Kovács[3], Henrietta Vadászi[3], András Micsonai[1,3], Melinda E. Tóth[7], Miklós Sántha[7], László Homolya[4], László Drahos[6], Gábor Juhász[2,8], Katalin A. Kékesi[1,2,9,*], and József Kardos[1,3,*,†]

[1]ELTE NAP Neuroimmunology Research Group, Department of Biochemistry, Institute of Biology, ELTE Eötvös Loránd University, Budapest, Hungary

[2]Laboratory of Proteomics, Institute of Biology, ELTE Eötvös Loránd University, Budapest, Hungary

[3]Department of Biochemistry, Institute of Biology, ELTE Eötvös Loránd University, Budapest, Hungary

[4]Molecular Cell Biology Research Group, Institute of Enzymology, Research Center for Natural Sciences, Hungarian Academy of Sciences Centre of Excellence, Budapest, Hungary

[5]Department of Biophysics and Radiation Biology, Semmelweis University, Budapest, Hungary

[6]MS Proteomics Research Group, Institute of Organic Chemistry, Research Centre for Natural Sciences, Budapest, Hungary

[7]Institute of Biochemistry, Biological Research Centre, Szeged, Hungary

[8]CRU Hungary Ltd., Göd, Hungary

[9]Department of Physiology and Neurobiology, Institute of Biology, ELTE Eötvös Loránd University, Budapest, Hungary

[*]These authors contributed equally to this work.

[†]Corresponding author, e-mail: kardos@elte.hu, phone: +36 1 411 6500 ext. 1795

Supplementary Table 1. List of proteins showing different levels in C1q-tagged synaptosomes between APP/PS1 and wild-type mice.[a]

Spot #	*P*-value	Fold change (APP/B6)	Protein name	Gene name
1	0.048	-1.35	Alpha-centractin	Actr1a
			Dynamin-1	Dnm1
			Dual specificity mitogen-activated protein kinase kinase 2	Map2k2
2	0.033	1.15	NSFL1 cofactor p47	Nsfl1c
			Actin, cytoplasmic 1	Actb
3	0.0049	-1.71	14-3-3 protein epsilon	Ywhae
			Tropomyosin alpha-1 chain	Tpm1
			Tropomyosin alpha-3 chain	Tpm3
4	0.031	-1.38	MICOS complex subunit Mic19	Chchd3
			Adenylate kinase 4, mitochondrial	Ak4
			Hydroxyacylglutathione hydrolase, mitochondrial	Hagh
			Protein lin-7 homolog A	Lin7a
			Enoyl-CoA hydratase, mitochondrial	Echs1
5	0.014	-1.63	Prohibitin	Phb
6	0.02	-1.35	Triosephosphate isomerase	Tpi1
7	0.012	-1.59	Prohibitin	Phb
8	0.029	-1.27	Protein-L-isoaspartate(D-aspartate) O-methyltransferase	Pcmt1
			Triosephosphate isomerase	Tpi1
9	0.014	-1.64	ATP synthase subunit d, mitochondrial	Atp5h
10	0.029	1.6	Peroxiredoxin-2	Prdx2
			Phosphatidylethanolamine-binding protein 1	Pebp1
11	0.028	-1.58	Cytochrome c oxidase subunit 5A, mitochondrial	Cox5a
12	0.045	-1.72	Cytochrome c oxidase subunit 5A, mitochondrial	Cox5a
			Thioredoxin	Txn
13	0.044	1.53	Alpha-enolase	Eno1
14	0.033	-1.4	Voltage-dependent anion-selective channel protein 1	Vdac1
			Voltage-dependent anion-selective channel protein 2	Vdac2
15	0.0043	-1.43	Dihydropyrimidinase-related protein 2	Dpysl2
			Actin, cytoplasmic 1	Actb
			Serine-threonine kinase receptor-associated protein	Strap

[a]Green color indicates mitochondrial and energy metabolism-related proteins.

Supplementary Table 2. List of proteins identified in APP/PS1 mice showing different levels between C1q-tagged and untagged synaptosomes.[a]

Spot #	*P*-value	Fold change (C1q-tagged /untagged)	Protein name	Gene name
1	0.0004	1.28	Heat shock 70 kDa protein 4L	Hspa4l
2	0.0293	1.20	Heat shock 70 kDa protein 4L	Hspa4l
3	0.0316	1.14	Dynamin-1	Dnm1
			Programmed cell death 6-interacting protein	Pdcd6ip
4	0.0181	1.18	Dynamin-1	Dnm1
			Programmed cell death 6-interacting protein	Pdcd6ip
5	0.0252	1.18	Dynamin-1	Dnm1
6	0.0237	1.16	Programmed cell death 6-interacting protein	Pdcd6ip
			Dynamin-1	Dnm1
7	0.0343	1.33	Synapsin-1	Syn1
8	0.0085	-1.20	Dihydrolipoyl dehydrogenase, mitochondrial	Dld
9	0.0210	-1.19	Pyruvate kinase PKM	Pkm
10	0.0474	-1.16	Pyruvate kinase PKM	Pkm
11	0.0445	1.15	Fascin	Fscn1
			Aldehyde dehydrogenase X, mitochondrial	Aldh1b1
			Inosine-5'-monophosphate dehydrogenase 1	Impdh1
12	0.0015	-1.29	Septin-4	Sept4
13	0.0026	-1.35	Dihydrolipoyllysine-residue succinyltransferase component of 2-oxoglutarate dehydrogenase complex, mitochondrial	Dlst
			Septin-4	Sept4
14	0.0060	1.21	Septin-11	Sept11
15	0.0387	1.25	Glutamate dehydrogenase 1, mitochondrial	Glud1
16	0.0354	-1.43	Tubulin beta-5 chain	Tubb5
			Tubulin beta-2A chain	Tubb2a
			Tubulin beta-4B chain	Tubb4b
			Tubulin beta-3 chain	Tubb3
17	0.0231	-1.39	Alpha-enolase	Eno1
18	0.0167	1.33	Septin-7	Sept7
19	0.0221	-1.17	Alpha-enolase	Eno1
20	0.0209	1.41	Pyruvate dehydrogenase E1 component subunit alpha. somatic form, mitochondrial	Pdha1

21	0.0438	-1.22	Fructose-bisphosphate aldolase A	Aldoa
22	0.0072	1.27	Neuronal-specific septin-3	Sept3
23	0.0094	3.24	Neuronal-specific septin-3	Sept3
24	0.0094	1.68	Neuronal-specific septin-3	Sept3
			Septin-5	Sept5
25	0.0017	1.39	Malate dehydrogenase, cytoplasmic	Mdh1
			Neuronal-specific septin-3	Sept3
26	0.0308	1.11	Transaldolase	Taldo1
27	0.0067	-1.32	Monoglyceride lipase	Mgll
			Voltage-dependent anion-selective channel protein 2	Vdac2
28	0.0003	-1.38	L-lactate dehydrogenase A chain	Ldha
29	0.0165	1.31	Malate dehydrogenase, mitochondrial	Mdh2
30	0.0058	1.17	Ribose-phosphate pyrophosphokinase 1	Prps1
31	0.0003	-1.17	Malate dehydrogenase, cytoplasmic	Mdh1
32	0.0044	1.23	Prohibitin	Phb
33	0.0028	2.28	Carbonic anhydrase 2	Ca2
			Phosphoglycerate mutase 1	Pgam1
34	0.0001	1.52	Hydroxyacylglutathione hydrolase, mitochondrial	Hagh
			Proteasome subunit alpha type-4	Psma4
35	0.0391	1.80	ES1 protein homolog, mitochondrial	D10Jhu81e
			Glutathione S-transferase Mu 1	Gstm1
			Triosephosphate isomerase	Tpi1
36	0.0201	1.54	NADH dehydrogenase [ubiquinone] flavoprotein 2, mitochondrial	Ndufv2
37	0.0030	1.24	Ras-related protein R-Ras2	Rras2
38	0.0082	1.24	ATP synthase subunit d, mitochondrial	Atp5h
39	0.0002	1.26	Cytochrome c oxidase subunit 5A, mitochondrial	Cox5a
40	0.0072	1.18	Proteasome subunit beta type-2	Psmb2
41	0.0436	-1.12	Acyl-protein thioesterase 2	Lypla2
			Mitochondrial peptide methionine sulfoxide reductase	Msra
42	0.0434	-1.41	Syntaxin-binding protein 1	Stxbp1
			Fascin	Fscn1
			Dihydrolipoyl dehydrogenase, mitochondrial	Dld
43	0.0037	-1.54	Tubulin beta-2A chain	Tubb2a
			Tubulin beta-5 chain	Tubb5
			Tubulin beta-4B chain	Tubb4b

			Tubulin beta-4A chain	Tubb4a
			Actin, cytoplasmic 1	Actb
44	0.0003	-1.18	Tubulin beta-4A chain	Tubb4a
			Tubulin beta-4B chain	Tubb4b
			Tubulin beta-5 chain	Tubb5
			Tubulin beta-2A chain	Tubb2a
			Actin, cytoplasmic 1	Actb
45	0.0071	-1.44	Ubiquitin-conjugating enzyme E2 K	Ube2k
46	0.0373	1.27	Actin, cytoplasmic 1	Actb
			Histidine triad nucleotide-binding protein 1	Hint1

[a]Green color indicates mitochondrial and energy metabolism-related proteins. Identified proteins of the septin family are shown in orange.

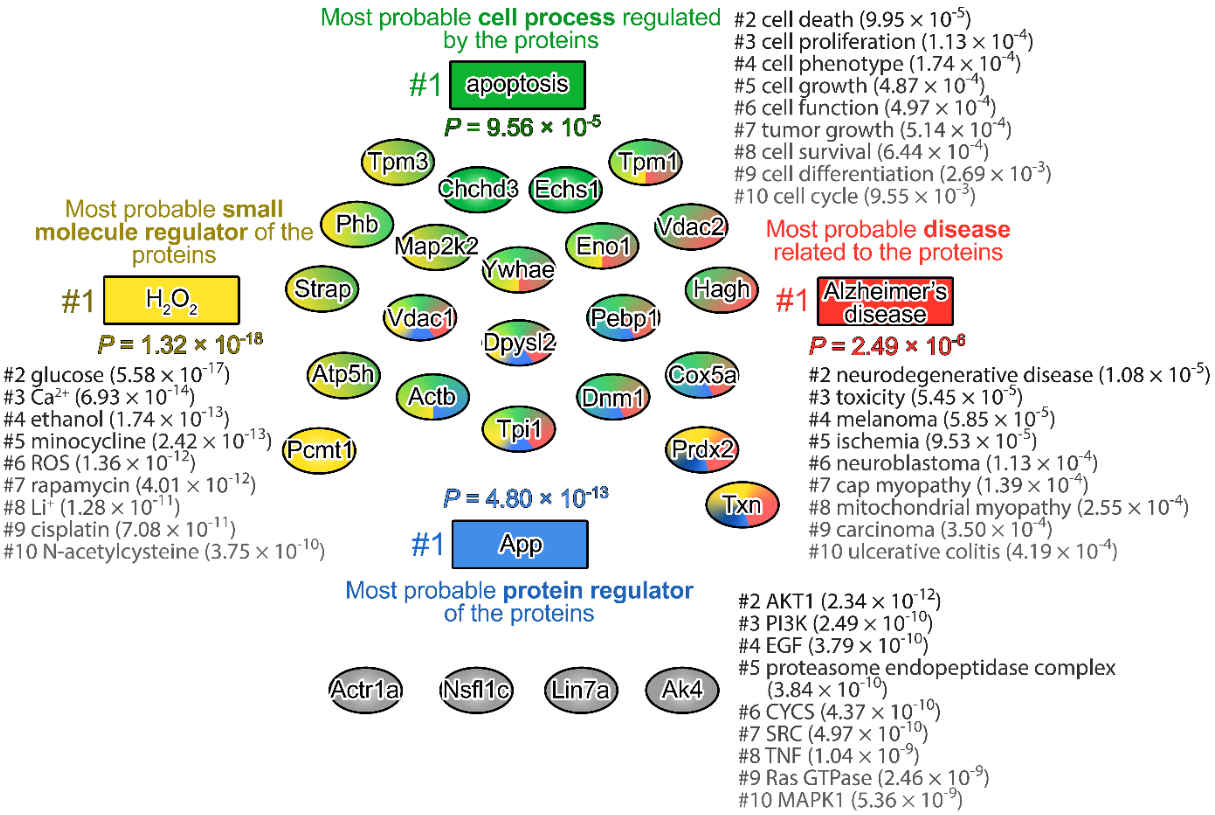

Supplementary Fig. 1. Enrichment analysis results of the altered proteins (in ellipses) identified in study #1. The most significantly enriched regulators/regulated entities are depicted proposed by using the Pathway Studio software. Proteins are colored according to the entities they are linked to at first rank of the corresponding categories. Identified proteins in grey ellipses showed no association with the entities. Statistically significant enrichment was determined using Fisher's exact test.

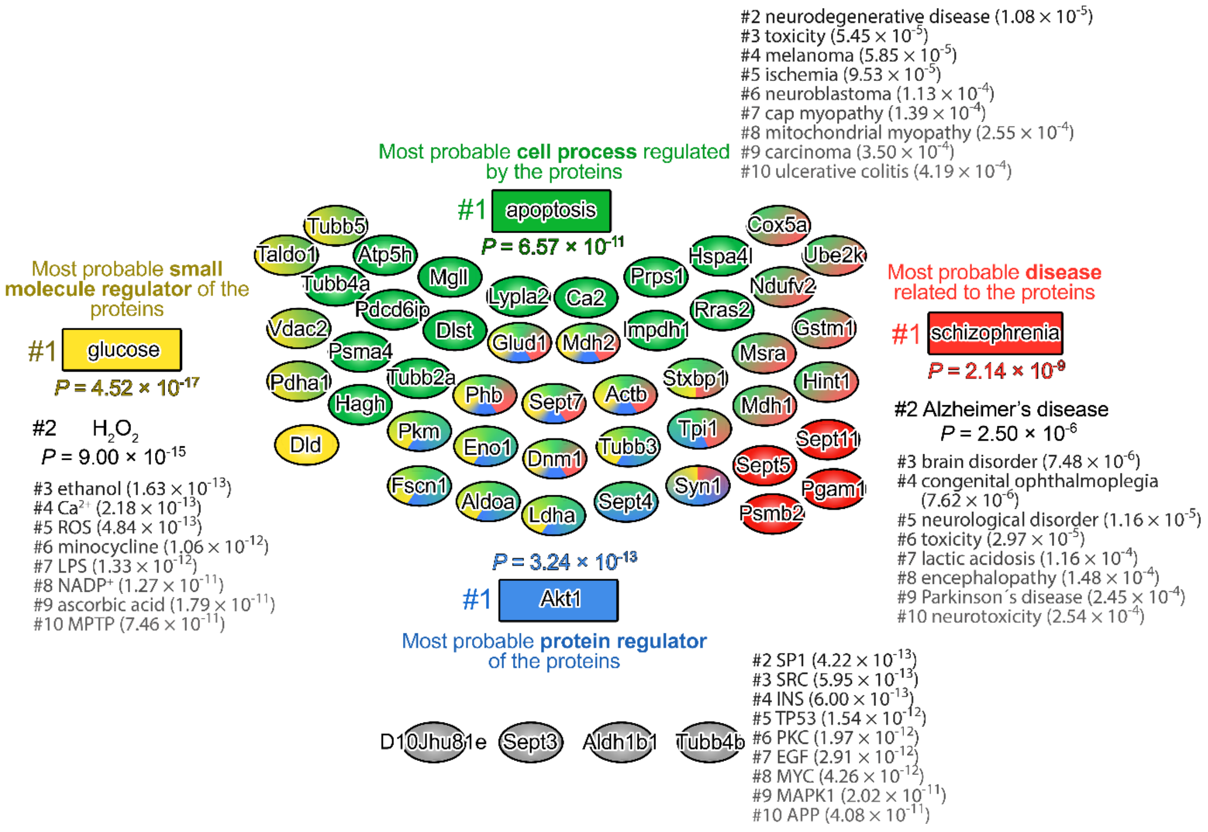

Supplementary Fig. 2 Enrichment analysis results of the altered proteins (in ellipses) identified in study #2. The most significantly enriched regulators/regulated entities are depicted, which were proposed by using the Pathway Studio software. Proteins are colored according to the entities they are linked to at first rank of the corresponding categories. Identified proteins in grey ellipses showed no association with the entities. Statistically significant enrichment was determined using Fisher's exact test.

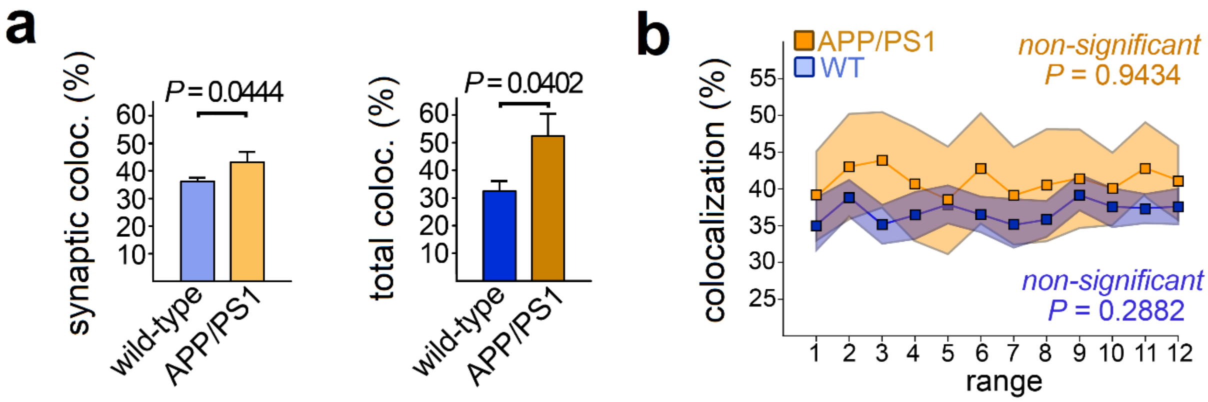

Supplementary Fig. 3 C1q accumulation in APP/PS1 mice is also linked to Sept5 levels as demonstrated by immunostaining of cerebral cortical brain sections. **a** Bar graphs show a statistically significant elevation in the percentage of synaptic and total Sept5 that colocalize with C1q in APP/PS1 mice compared to the wild-type ones. **b** Line graph demonstrates the correlation between the percentage of synaptic C1q-colocalized Sept5 and Sept5 levels. Ranges were defined as described in Fig. 5c. At an alpha level of 0.05, we did not observe a statistically significant correlation between the investigated variables in neither of the mouse strains. Means ± S.E.M. are shown. *n* = 4-6 images from 2-3 animals per mouse strain. Two-tailed Student's *t*-test of independent samples was used for the comparisons. Pearson's correlation coefficient (*R*) and the two-tailed *P* value were determined for the correlation analyses.
